# Reducing long‐term use of benzodiazepine receptor agonists: In‐depth interview study with primary care stakeholders

**DOI:** 10.1111/hex.13888

**Published:** 2023-10-17

**Authors:** Kristien Coteur, Sanne Peters, Pieter Jansen, Birgitte Schoenmakers, Marc Van Nuland

**Affiliations:** ^1^ Department of Public Health and Primary Care Academic Center for General Practice, KU Leuven Leuven Belgium; ^2^ Division of Medicine, Dentistry and Health Sciences School of Health Sciences, The University of Melbourne Melbourne Australia

**Keywords:** benzodiazepines, deprescriptions, general practitioners, grounded theory, medication therapy management, pharmacists, primary health care

## Abstract

**Aims:**

To increase our understanding of which factors contribute to long‐term benzodiazepine receptor agonist (BZRA) use for insomnia in primary care, from a patients', general practitioners' (GP) and pharmacists' perspective.

**Design:**

Qualitative research following a grounded theory approach.

**Setting:**

Primary care in Belgium.

**Participants:**

Twenty‐four participants were interviewed, including nine patients, six GPs and nine pharmacists.

**Measurements:**

In‐depth, semistructured interviews with iterative cycles of data collection and analysis. Transcripts were analysed using the framework method. Thematic findings were interpreted in the context of the Theoretical Domains Framework.

**Findings:**

A reflexive relation was identified between views about hypnotic use at the level of society, healthcare and patients. Behaviour change appeared to depend strongly on context and social influence, including a need for supporting relationships by all stakeholders. Six key messages captured factors that contribute to long‐term BZRA use for insomnia in primary care: societal beliefs as a game changer, the opportunity of nonpharmacological treatment, collaborative primary care, patient‐centred goals, informed consent and self‐management.

**Conclusions:**

Long‐term BZRA use for insomnia is a complex and multifaceted public health problem that is not adequately addressed in primary care at this time. Although primary care professionals in this study found discontinuation of long‐term BZRA use relevant to the patient's health, many organisational and personal barriers were reported. Moreover, the current social and healthcare context is not empowering patients and professionals to reduce long‐term BZRA use for insomnia. Specifically, for primary care, all stakeholders reported the need for a nonmedicalised relationship between the patient and GP to lower prescribing rates.

**Patient or Public Contribution:**

The Flemish Patient Platform, a patient representative organisation, assisted with recruitment by launching a call for participants in their newsletter and volunteered to disseminate the results. The call for recruitment was also published online in social media groups regarding insomnia and via posters in public pharmacies. Patients or public were not involved in designing or conducting the interview study.

## 1 INTRODUCTION

Long‐term use of benzodiazepine receptor agonists (BZRAs) remains a common public health problem that puts patients at risk of memory, psychomotor and cognitive impairment.^1^, ^2^ Although it has often been associated with addiction,^3^, ^4^, ^5^ most primary care patients use their BZRA as prescribed, often maintaining a steady dose and without signs of craving.^4^, ^6^, ^7^ When discontinuing long‐term use, psychological or functional decline are possible,^3^ and are likely to relate to withdrawal symptoms because of physical dependence.^7^ Physical dependence is common, and presumed to play an important role in persistent use.^8^ Therefore, a slow taper protocol and structural follow‐up are recommended, as advised in the National Institute for Health and Care Excellence guidelines on safe prescribing and withdrawal management of medicines associated with dependence.^9^

In Belgium, long‐term BZRA use is especially common for insomnia although primary care treatment guidelines recommend a stepped care approach, with medication being reserved for acute situations with a high burden of suffering for the patient.^10^, ^11^ When started, BZRA prescriptions should be accompanied by patient education about possible therapeutic and adverse effects, and a plan for discontinuation. Ideally, BZRAs are used with the lowest possible dose for the shortest possible duration, and a follow‐up appointment with the general practitioner (GP) is scheduled after 1 week.^9^, ^10^ However, some barriers to the appropriate use of BZRAs in primary care have previously been reported. GPs have described a lack of time and funding.^12^, ^13^ They find it difficult to manage BZRA use while preserving a good GP–patient relationship.^13^ The older age of patients is viewed as a reason not to initiate or discuss discontinuation.^12^ There is a lack of knowledge and services to manage complex cases of insomnia or BZRA withdrawal.^13^ Many patients believe that they need their BZRA for everyday coping and functioning,^14^, ^15^ and perceive other strategies as ineffective.^14^ Patients continue BZRA use because they feel that they are unable to stop, fear the consequences of stopping or cannot tolerate the withdrawal symptoms,^14^, ^15^ such as rebound insomnia. They often believe that the benefits outweigh the risks, which attests to a lack of information and discussion about discontinuation and alternative treatment options.^12^, ^15^ Patients passively trust their GP, and many have been reassured about the safety and necessity of BZRA use by a physician.^14^, ^15^ Besides remediating these barriers, continuity of care and taking patients' problems seriously were considered facilitators to the appropriate use of BZRA.^14^, ^15^

Belgium remains among the top three countries with most licit BZRA use worldwide.^16^ Despite repetitive national campaigns aimed at education and sensitisation of both primary care providers and patients in the past 20 years, no noteworthy changes in prescriptions and long‐term use have been found, with an estimated prevalence of 5% among the adult population.^11^, ^17^ To inform future action plans, this project aims to increase our understanding of all factors that currently contribute to long‐term BZRA use for insomnia in primary care, from patients', GPs' and pharmacists' perspectives.

## 2 METHODS

### 2.1 Study design and setting

Given the complex relation between society, primary care and patients' behaviour, qualitative methods are best suited to study BZRA use with a real‐world population.^18^ When applied with scientific rigour, qualitative research has the potential of leading to novel policy and interventions for reducing long‐term BZRA use in primary care. In‐depth interviews are ideal to obtain an overview of barriers and enablers because they allow deviation from the topic guide to thoroughly explore the interviewee's narrative,^19^ including needs and context factors that might contribute to long‐term BZRA use.

This qualitative cross‐sectional study in Flanders, Belgium, followed a grounded theory approach.^20^ Grounded theory aims to understand social processes that influence a certain phenomenon.^20^ In the process, theoretical patterns are developed based on the data and relevant theory.^21^ Ethics approval was obtained from the Social and Societal Ethics Committee of KU Leuven in August 2021 (G‐2021‐3713‐R2(MAR)).

### 2.2 Sampling and recruitment

For data source triangulation, we recruited a convenience sample of three key stakeholders in primary care, namely patients, GPs, and pharmacists, who are respectively the users, and providers of BZRAs.^22^ Adult (18+), community‐dwelling patients with current or previous BZRA use for insomnia living in Belgium were reached via their GP, pharmacist or online patient fora with a flyer or advertisement containing a QR code for a registration form. Next, we contacted patients via e‐mail to provide more information and verify their interest in participating. Primary care professionals (PC‐Profs), including both GPs and pharmacists, were reached via professional associations, the researchers' network and personal contact. If respondents were interested, we e‐mailed them the informed consent form and scheduled the interview. Recruitment continued until data saturation was confirmed.^23^

#### 2.2.1 Participant characteristics

In total, we conducted 24 interviews between September 2021 and April 2022. Participants' age varied from 29 to 78 years old (median 45), with 71% being female. More detailed characteristics are in Table 1. Nine PC‐Profs declined participation upon personal contact, because of a busy schedule (*n* = 5), without reason (*n* = 3) or lack of interest (*n* = 1). Due to the recruitment strategy, reasons for patients' nonparticipation could not be tracked.

**Table 1:** Background characteristics of participants.

Patients	(*n* = 9)
Age (years)	
Median (range)	52 (31–78)
Sex	
Female	8
Male	1
Duration of BZRA intake (years)	
Median (range)	7 (0.5–25)
Status of use	
Current	6
Previous	3
Co‐morbidity	
Anxiety	2
Cancer	1
Depression	2
Ehler–Danlos syndrome	1
Chronic pain	1
Multiple sclerose	1
Fibromyalgia	1
Concomitant medication use	
Antidepressants	4
Antipsychotics	2
Opioids	2
Melatonin	2

**Primary care professionals**	**(*n* = 15)**
Age (years)	
Median (range)	39 (29–63)
Sex	
Female	9
Male	6
Role	
General practitioner	6
Pharmacist	9

Abbreviation: BZRA, benzodiazepine receptor agonist.

### 2.3 Data collection

Semistructured interviews, via video‐call or in‐person, were conducted by K. C., a female researcher with interview experience in clinical and research settings. Privacy was guaranteed as only the interviewer and interviewee were present.^24^ The interviewer had no relationship with patients and pharmacists before the interviews, whereas some GPs may have been acquainted with the interviewer's research focus through previous communications in the university alumni journal, and two out of six (GP1 and GP2) had previously met K. C. during informal events at the university. Interviews, both transcripts and the interview context as experienced by the interviewer, were intermittently discussed in team with one junior (P. J.) and two experienced qualitative researchers (M. V. N., S. P.). These discussions helped evolve the topic guide (Table 2), along with multiple iterations of data collection and analysis.

**Table 2:** Summary of the topic guides and their adaptations.

	Patients	General practitioners and pharmacists
First focus	Narrative	BZRA management in daily practice with case vignettes
	Sleep	
	Hypnotics use	
Second focus	BZRA management	Multidisciplinary aspects of BZRA management in primary care
Third focus	Themes of previous research, e.g., peer support, health communication, motivation	A professionally perfect world (utopia)
Added foci	Phytotherapy	Reasons to start a discussion on BZRAs with patients/clients
		Generational problem
Adaptations control interviews	/	No case vignettes, but testing earlier findings
		Complex patient situations, patient empowerment
		Brief interventions
		First prescription or approval of renewal by specialist

Abbreviation: BZRA, benzodiazepine receptor agonist.

Interviews started after written informed consent and a personal introduction. With patients, we first focused on their narratives concerning sleep and hypnotic use. Then discussed their views on BZRA management and themes identified in previous research, such as peer support, health communication and motivation.^6^ With PC‐Profs, we first discussed BZRA management in daily practice starting from two case vignettes (Table 3), an easy and valid tool to explore quality of care.^25^ We continued with multidisciplinary aspects, utopic ideas and simultaneously tested findings from previous research.^6^, ^26^ Interviews lasted between 45 and 110 min (mean [SD]: 76′[21]′). We audio‐recorded all interviews and sent their verbatim transcription to the respective participant for review, following the Consolidated Criteria for Reporting Qualitative Studies (COREQ) guidelines.^27^

**Table 3:** Case vignettes.

Case vignette 1	Case vignette 2
Luk is a patient you have known for about 5 years. He is 42 years old and healthy, happily married, and lives 10 min from your surgery (pharmacy). Last week, his wife and daughter experienced a serious car accident. Fortunately, they were discharged from the hospital after a few days under observation. They are now back home to recover, and are expected to be able to do so fully. Since the accident, Luk has had problems sleeping. (Luk went to the GP for this and got a prescription for lormetazepam).	Marie is 64 years old and has been retired since last year. She is one of your regular patients (clients). Her previous GP prescribed her Temesta (lorazepam) for the first time when her husband had died suddenly. Marie was 48 years old at the time. Eight years ago, she switched to Zolpidem. Over the years, her use has fluctuated a bit, from 0.5 to 2 pills a day. Currently, she uses 1 pill a day.

Abbreviation: GP, general practitioner.

### 2.4 Data analysis

Pseudonymised transcripts were analyzed with the framework method.^28^ After familiarisation with the interviews, we used open coding, which is commonly associated with grounded theory, to inductively identify codes and themes.^29^, ^30^, ^31^ Each team member (K. C., S. P., M. V. N., P. J.) coded two interviews in NVivo12,^32^ eight in total, representing all participant groups. Findings from this first round of coding were combined by K. C. into a preliminary coding tree, which was then discussed in team. Next, all interviews were coded by a duo of the team, independently. Refinement of the coding tree (appendix 1) and anomalies were agreed upon by consensus. In our team discussions, we combined induction with abduction^31^, ^33^ to move beyond a mere description of participants' views and increase our understanding of the complex interplay of their needs and context factors. An iterative cycle of analysis and data collection continued until no new insights were found in the data.^23^ Two control interviews with PC‐Profs were conducted for confirmation.

In the next phase, the Theoretical Domains Framework (TDF)^34^ was used because it offers a theory‐based approach for identifying barriers and enablers. K. C. charted the data into a matrix based on the TDF, creating an overview of barriers and enablers for appropriate use of BZRAs that surpasses individual narratives. The matrix (Table 4) was verified by all team members, and specifically discussed with S. P., an implementation scientist who has experience using the TDF. Given the TDF ‘provides a theoretical lens through which to view the cognitive, affective, social and environmental influences on behaviour^35^ ^,p.2^, this mapping activity enabled us to identify key messages concerning the factors that contribute to long‐term BZRA use for insomnia in primary care.

**Table 4:** Themes mapped to TDF domains per participant group, with commonalities centralized (needs are reported in regular font, while context factors are reported in italics).

TDF domains	Patients	Primary care professionals
Knowledge	Information on drug (side) effects	
	Information on alternative treatment and discontinuation options (education/training)	
	Expertise of GP/pharmacist to start conversation on BZRA use	
Skills		*Not trained to treat psychopathology*
	Treatment/discontinuation tailored to the patient	
Social/professional role and identity	in need of rest (vs. being able to hear partner at night)	*(Not) feeling personally responsible for overconsumption*
	*Views on psychotherapy*	*Tacit acceptance (steady use is prolonged)*
	*Prescribing physician responsible for overconsumption*	
		Providing similar advice (also across disciplines)
		*Patient decides (sometimes pressures GP/pharmacist)*
Beliefs about capabilities	Something to rely on for when they feel particularly stressed, a comfort	External trigger to start conversation on long‐term BZRA use
Optimism	–	–
Beliefs about consequences	*Tacit acceptance (steady use is prolonged)*	
	Discuss medication interactions	
Reinforcement	Follow‐up (patient: best treatment for current situation; GP/pharm: prescribing and selling behaviour, audit, etc.)	
		*Low return on investment (when trying to work nonpharmacologically or discontinue)*
Intentions	Something to rely on (doing well with hypnotics, not willing or afraid to adjust)	Providing help in acute situations
		*Fall prevention: switch medication*
Goals	Functioning during the day (life adjustments; closely related to stressors)	Patient in best possible health
	Nonmedicalised relationship between patient and GP	
	Be a normal human again, without pills	Good relationship with patient
Memory, attention and decision processes	Input decision to use BZRAs	
Environmental context and resources	*Access to psychotherapy (sessions limited vs. tailored approach; waiting lists; financial investment)*	*Misuse accepted (harmful prescribing) (=denial of patients’ capabilities to reduce or stop)*
		*No priority because one of many public health worries*
		*Pharmacist is obliged to sell full box of what is prescribed to patient*
		*Lack of network to refer to for addiction care*
		*Started, sometimes stopped in hospital or residential care*
	Financial (cheapest solution)	Financial (voluntary time investment)
		Patient motivated to reduce or stop
		Self‐management by patient
		Effective alternative treatment options
		Improved working conditions (for medication reviews; for psychosocial consultations)
	Group sessions	
	Online tools	
	Privacy and safe environment to start conversation on BZRA use	
Social influences	Social support	*Lack of interdisciplinary communication (pharmacist may not know indication for use)*
		*Prescribing physician has the power (GP > pharmacist; specialist* > *GP)*
		*Responsibility pharmacist in case of harmful prescribing (will work with patient without physician if necessary)*
		Good work relationship between GP and pharmacist
	*Long‐term users viewed as addicts*	
	*Fundamental societal problem of stress (management)*	
	Less stigma	
	*(No) taboo*	
	Respectful communication (i.e., recognition of complexity) to start conversation on BZRA use	
Emotion	Something to rely on (because of deep sadness or complex situation)	
Behavioural regulation		*BZRAs sold without valid prescription*

Abbreviations: BZRA, benzodiazepine receptor agonist; GP, general practitioner; TDF, Theoretical Domains Framework.

We report our findings following the standards for reporting qualitative research^36^ and COREQ.^27^ Short excerpts of the interviews, such as single words or short statements, and longer excerpts are presented as examples of the findings. We briefly report important deviant views to demonstrate that themes represent reoccurring patterns rather than collective beliefs.

## 3 RESULTS

**Figure 1:**
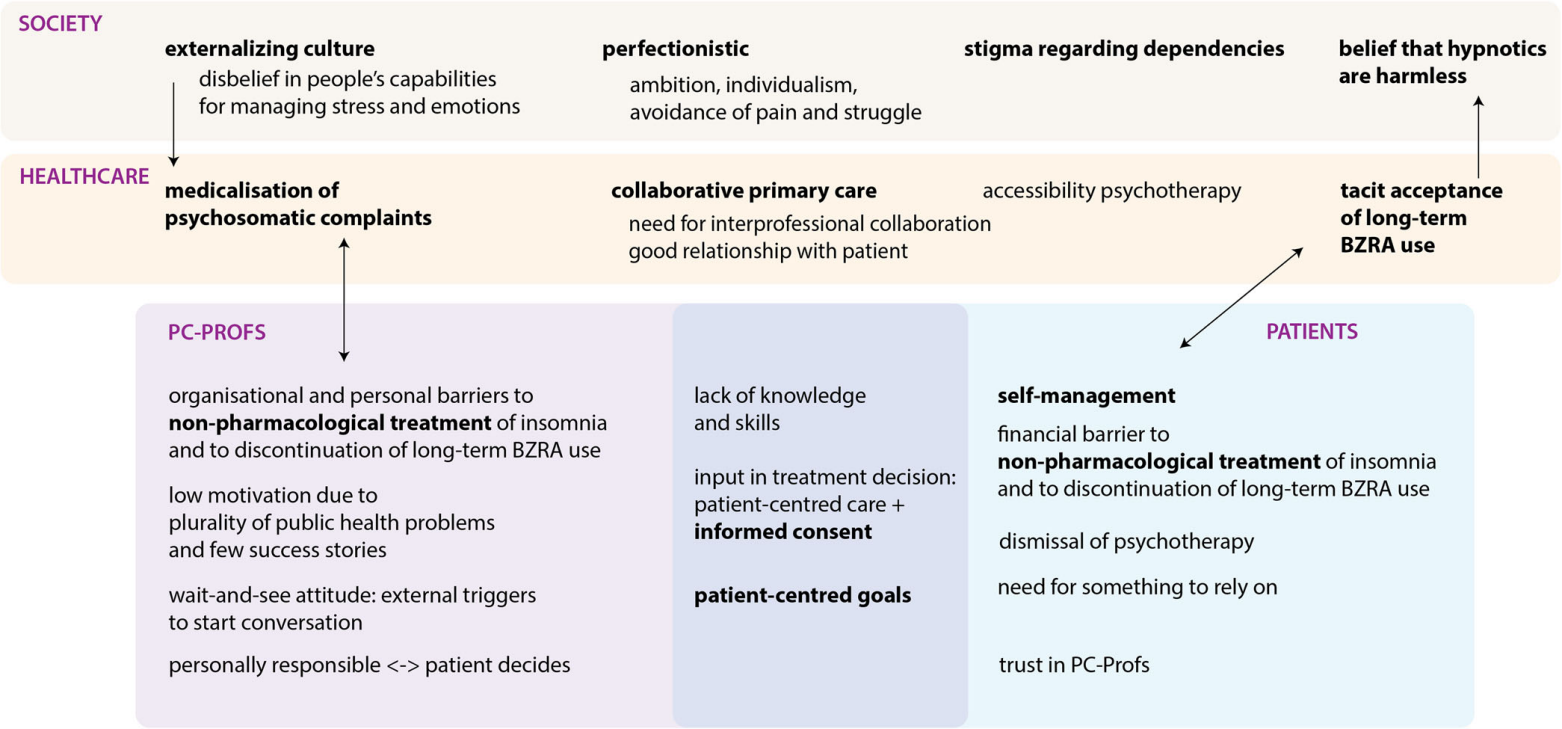
Overview of factors that contribute to long‐term benzodiazepine receptor agonist (BZRA) use for insomnia in primary care at the level of society, healthcare, professionals (PC‐Profs) and patients. Themes common to both PC‐Profs and patients are in the middle (purple box). Core themes are in bold. The arrows emphasise some reinforcing relationships, illustrating the reflexive relation between views about hypnotic use at different levels.

Overall, the analysis revealed the reflexive relation between views about hypnotic use at the level of society, healthcare and patients (Figure 1). This is a complex connection that indirectly steers behaviour. Behaviour change with regard to BZRA use appeared to depend heavily on context and social influence, including a need for supporting relationships by all stakeholders. The main findings are summarised in six key messages, integrating barriers and enablers from multiple TDF domains (Table 4).

### 3.1 Societal beliefs as a game changer

Participants reported some deep‐rooted beliefs in society that contribute to long‐term BZRA use. First, the public believes that hypnotic medication use is harmless (TDF domain social influence). This was reflected in the common practices of patients recommending or passing along their medication to peers, and referring to the drugs with diminutive wording.

Only one pill is being made cute about, and that's the sleeping pill. That's just a Temesta'ke [lorazepam] or Stilnoct'je [zolpidem]. ['ke and 'je create diminutives in Dutch.] (GP3)

The beforementioned belief is reinforced by the tacit acceptance of steady long‐term use by PC‐Profs (TDF domains beliefs about consequences and social/professional role and identity).

I think that they are just convinced that it works, so why stop? And the doctor prescribes it, and the pharmacist says nothing about it, yes. (Ph6)

Second, participants reported that medicalisation of psychosomatic complaints is generally accepted. It appears to be reinforced by an individualistic and perfectionistic culture (TDF domain social influences and environmental context and resources).

When people go to work that pressure is so high. The children are there, there are many… young families who have built or bought their house, they can't stop working because everything has to be paid for. … The pressure in people is so high and they want to participate in as many things as possible that they live more alongside their bodies than inside them. And that, that stress that…that backpack that they lug around every day… Makes them unable to sleep at night, which makes that yes that…all that information and those triggers in their head is so high that their mind finds no, no rest anymore, I think. (patient 6)

Third, although both patients and PC‐Profs desired a nonmedicalised relationship (TDF domain goals), there is little belief in people's own capabilities to overcome psychosocial problems. Additionally, hypnotic use is perceived as a ‘quick fix’ that is more accessible than nonpharmacological treatment of insomnia (TDF domain environmental context and resources).

Finally, some participants reported stigma because of the dependency‐aspect, while others reported no stigma at all. Their different experiences aside, the common belief was that it should not be taboo to discuss long‐term BZRA use (TDF domain social influences).

The stigma is ‘he's going to start talking about it again’, that is why it comes at the end [of the consultation]. I think many [patients] notice that they have a problem. (GP0)

—The shame you talk about, where does that come from?—Euhm…Well, that I have been using those benzos for so long… (patient 5)

Um, what I do find more difficult to talk about is the addictive effect of the medication or, um, I would tell my doctor, ‘Yes, I really notice that it is addictive and I am also taking more…’, and I find that more difficult to discuss, so, um, I would leave it out or I would go straight to the psychologist… It's really like sharing something that's not allowed. (patient 0)

### 3.2 The opportunity of nonpharmacological treatment

Nonpharmacological treatment of insomnia was viewed as an expensive and tiresome investment. PC‐Profs reported a lack of time or renumeration for their invested time when preventing BZRA use or reducing long‐term use (TDF domain environmental context and resources).

What could really support me is to provide enough time that we get funding to phase things out, because…you know…it costs me and also yes to [use] those non‐pharmacological [interventions], that means running 25 minutes late anyway. If I just prescribe something, it takes me 5 minutes. […]I don't want to prescribe that to my patients, I have never prescribed it. But to not prescribe it, I have to put a lot of time and energy into it and [I would like] that I am remunerated for time and energy. (GP5)

Their motivation to work nonpharmacologically also fluctuated because of the plurality of public health problems that they prevent or treat (TDF domain environmental context and resources), and the relatively low number of success stories (TDF domain reinforcement).

Patients reported financial means as an important barrier to specialised care (TDF domain environmental context and resources). Additionally, psychotherapeutic treatment was dismissed by some patients (TDF domain social role and identity), not accessible due to high demand, or the PC‐Profs' professional network lacked appropriate therapists for adequate referral (TDF domain environmental context and resources).

Insomnia, there are some therapists who work with sleep but there are definitely not many and certainly not where I am GP. So then I'm kind of groping in the … yeah. Pff, I find it complex. (GP2)

Group sessions or online therapy were generally considered to be a valid alternative if they involved peer‐to‐peer contact and success stories (TDF domain social influences).

S…what might help…sleeping groups, sharing experiences. That might be something, because people now, you know online as well, it is also shared, those stories. […] I can imagine that it could help patients to … exchange experiences and maybe also success stories. (GP5)

I would be open to that, because … you go to a group session and there are also different… people, each in their own way, each person present there is going to tell their story and you can sometimes take things with you…. And I would definitely go to that.[…] because you can always take things with you from such a session. (patient 1)

Finally, expertise regarding BZRA discontinuation was an important need for both patients and PC‐Profs, including knowledge and skills to properly inform and empower patients (TDF domains knowledge and skills).

I think a lot of GPs misunderstand sleeping pills. If their patient says that they want to stop they usually say ‘you won't succeed anyway’. … That is definitely my experience. I've had four of them withdrawing without their GP knowing. (Ph5)

Although generally the prescription is viewed as the GP's responsibility (TDF domain social/professional role and identity), and patients viewed follow‐up as a crucial part of high‐quality care (TDF domain reinforcement), a wait‐and‐see attitude was common among PC‐Profs, who often required an external trigger to discuss BZRA use. This need for an external trigger could reflect disbelief in one's own capabilities, a lack of optimism, or motivation (TDF domain beliefs about capabilities).

Usually in response to a fact, an event. Yes, something traumatic. Which indeed allows those to… To start probing for motivation for change. Another handle to possibly start talking about change is if they start talking about memory problems. (GP4)

### 3.3 Collaborative primary care

Supportive relationships were important to all stakeholders. First, for PC‐Profs, interprofessional communication was key to providing the best possible care to the patient (TDF domains professional role and identity and social influence).

First of all, there should be more communication between doctors and pharmacists …. communication between the two groups. Um, what are everyone's strengths, and how can we use them to counteract overconsumption. (Ph6)

Although most strive for a good relationship between the prescriber and provider (TDF domains professional role and identity and social influences), professional conflicts do arise, for example, by dispensing BZRAs without a prescription when ‘I used all my powers to get rid of it [BZRA use] for a year…I am not providing that prescription’ (GP0), or the prescriber refusing the pharmacist's advice on medication interactions.

Second, PC‐Profs prioritised a good relationship with the patient (TDF domain goals). Fear of affecting that relationship could induce hesitancy about interventions. Often, pharmacists preferred contacting the GP to discuss discontinuation (TDF domain social influences).

Yes then you generally say to the patient: ‘Look, is it all right if I talk to your doctor about this? And… Make an appointment with your doctor and then I ask the doctor to prescribe a tapering schedule, in such a way that the patient can stick to it, that they have regular feedback from the doctor’. (Ph2)

We only have influence to say [to the patient] ‘would you do this’, but then you have to be careful not to get in the way of…some GPs will blame you if you say that. There are those who are happy if you open your mouth, but there are also those who say, ‘Yes, I have decided that he can have it, and you are the pharmacist so…’ (Ph5)

PC‐Profs aimed to provide similar advice to patients (TDF domain professional role and identity), except when there was a conflict of interest, that is, possible harm to the patient. Then, one‐sided interventions, for example, without involving the GP, became acceptable (TDF domain social influences).

Yes, it is true, because I did have a patient who tapered down with me because I believed that it was getting out of hand, um, and the doctor just kept prescribing, but yes, that's such a doctor, yes, I don't call him anymore, um, yes, so that's, yes, for example, if you call there for a drug interaction, he says ‘there is no such thing’, ok, well, then we are just working on other things, so then I do it myself, so then I just do it without the doctor knowing, um, just with the patient. (Ph1)

This could be related to the professional identity of PC‐Profs, with some reporting a strong sense of personal responsibility for the misuse of BZRAs (TDF domain professional role and identity). Although not all feel personally concerned by the problem, participants put responsibility for the BZRA overconsumption on the prescriber (TDF domains social influences and professional role and identity).

Gosh, the doctor prescribes that, of course. Following what the patient tells … the doctor. So the overconsumption actually, I think, comes from the healthcare providers because that's them prescribing. But … the reason why they go to the doctor, is what I told before. Because there is too much work pressure, I can't sleep I have too much stress at work. (patient 7)

Maybe also the GPs more. I don't know… how that is, but with the GP lies the key of prescribing so I think if they are a bit better informed or can spare a bit more time to discuss that. (Ph7)

I think we are largely responsible for that. (GP4)

Third, professional collaboration might be blocked by different views concerning the appropriate use of BZRAs: for example, short‐term use could differ between ‘two or three nights’ (GP0) and ‘maximum one month’ (Ph7).

Fourth, although the prescriber is responsible for providing the patient medication, ultimately the patient was viewed as responsible for its use. Nevertheless, their use is impacted by empowerment, prescribing behaviour, interventions by PC‐Profs, and other context factors.

Especially me as a prescriber and him as a user. I think our roles there are equal. With misuse, there's nothing I can do about that ultimately. I can only warn him, that's all I can do. (GP4)

### 3.4 Patient‐centred goals

Goals were found to be an important driver for behaviour. PC‐Profs aimed to help patients obtain their best possible health, whereas patients mainly aimed to function during the day (TDF domain goals).

Then I have to do my explanation about the dangers, what types of medication, which I prefer to use if he asks to take medication and then we try to find a consensus there, because people want something that works and we don't want that, because that is poison. (GP0)

Although these goals do not directly contradict one another, patients often felt that they need BZRAs, while PC‐Profs view BZRAs as a short‐term solution. However, due to organisational and personal barriers for nonpharmacological treatment, and the lack of other ‘quick fixes’, BZRA use is often prolonged. Especially when the patient persists.

…it's also just easy to keep prescribing it and keep delivering it, for us too. I think the doctor contributes most to overconsumption because [it stops] if he says no I won't do it anymore. But nowadays those people just go to another doctor. We have people who go to 4/5 GPs. (Ph7)

What we do, a certain generation is already on it, we tolerate everything there. (GP0)

When patients become motivated for discontinuation, their goals change. Often, they no longer want to rely on medication for their daily functioning, which is an important indicator of a switch in beliefs about consequences and capabilities (TDF domains beliefs about capabilities and beliefs about consequences).

Okay, it happened, you lost your husband, but you have to go back to sleep naturally again. Yes, that has always been my goal. That I go back to being a normal human being, but without pills. (patient 1)

Finally, patient centredness is a key aspect of interventions regarding BZRA use because PC‐Profs feel that ultimately the patient decides (TDF domain social/professional role and identity). Patient‐centred strategies like tailoring treatment to the patient's situation were essential to all stakeholders (TDF domain skills). Important elements are structural follow‐up of the patient's situation and informed consent (TDF domains skills and knowledge).

### 3.5 Informed consent

Although patients rely on PC‐Profs' medical expertise, they also contribute to the treatment decision (TDF domain memory, attention and decision processes). Therefore, they require to be well informed about the treatment's potential risks and benefits, and about alternative options (TDF domain knowledge).

I decide in consultation with the GP. (patient 0)

Even though some patients were well informed at the start of their treatment, risks and benefits are rarely revisited when prolonging the treatment. This is mainly blocked by organisational and personal barriers of PC‐Profs, such as a lack of time, fear of harming the good relationship with the patient, or insufficient knowledge and skills.

You always have to assume, the fact that the patient takes it with them means the patient has to be informed of … that it actually has some risks and he may not feel those now, but he may have them in the future. I think that's our job. (GP5)

Additionally, although PC‐Profs reported that the patient ultimately decides, patients trust their healthcare provider to choose what is best for them.

Yes, I have no knowledge about that [which medication is right for me]. For that, I completely rely on my GP and pharmacist. (patient 6)

### 3.6 Self‐management

Self‐management by the patient was viewed as an important aspect for PC‐Profs' motivation to continue BZRA use.

At some point you really lose them, you have to provide those tools, that they get it. And then it's their responsibility. You can't live their life for them, can you? (GP1)

Even though I prescribe sleep medication, I say it does not absolve them of their ‘duty’—may be a harsh word—but their sleep hygiene should also be in order. (GP2)

However, adequate self‐managing behaviour was blocked in many ways. First, patients lacked knowledge of treatment options (TDF domain knowledge). Second, financial barriers, and accessibility as previously mentioned, limited their treatment options (TDF domain environmental context and resources). Third, dysfunctional beliefs about capabilities were reported at all levels (TDF domain beliefs about capabilities). Societal beliefs about people's capabilities to manage stress and emotions were influenced by a strong externalising culture (TDF domain social influences).

Perhaps, in Belgium, people are too quick to turn to sleep medication instead of addressing the cause. (patient 7)

I think people have to learn to deal with pain, and recovery. That it takes time. (Ph6)

Our healthcare system reinforces these beliefs by medicalising psychosomatic problems, as discussed previously. Patients have internalised these beliefs, which was expressed in their views on hypnotics as something to rely on, a comfort to look forward to in the evening, knowing they have an external solution for when they feel stressed (TDF domains emotion and beliefs about capabilities).

## 4 DISCUSSION

This qualitative study found that long‐term BZRA use for insomnia is steered by a complex interplay of factors on a societal, professional and individual level. Barriers to an appropriate use of BZRAs are mainly situated in the domains of environmental context and resources, and social influences. No themes could be mapped to the domain of optimism. Patients and PC‐Profs would benefit from more empowerment and knowledge regarding discontinuation of long‐term BZRA use.

Our findings are largely in line with research from other countries showing that both the GP and pharmacist play an important role in implementing interventions to reduce long‐term BZRA use.^14^, ^15^, ^37^, ^38^, ^39^ A recent survey revealed that BZRA‐using patients prefer to discuss alternative behavioural therapy with the GP or pharmacist.^40^ Nonetheless, we found that a lack of interprofessional collaboration and aiming to preserve a good relationship with the patient at all costs, interfered with opportunities for patient education and informed consent. Both are important for quality care because repetition improves patient recall, just like providing written material,^41^ for example, a discontinuation letter, and informed consent is a guiding principle in patient‐centred care.^42^ Previous research confirmed that although patients may be well informed at the time of prescription, half of the information is not remembered 2–3 weeks later.^43^ This reveals the importance of regularly re‐evaluating the patient's situation and rediscussing the risk‐benefit profile of long‐term BZRA use or suggesting a BZRA reduction, which has previously been described by patients as part of the duty of care of the prescriber.^44^

Long‐term prescribing counters the PC‐Profs' aim of helping patients obtain their best possible health because of BZRA side effects. Although some PC‐Profs believe that their patient needs BZRAs for sleep—because of the negative health effects of disturbed sleep^45^—there is no evidence of long‐term efficacy.^46^ Conversely, there is evidence that suggests that quality of life worsens in patients who continue or restart BZRA use in comparison to patients who stopped their intake.^38^

Although effective brief interventions for deprescribing of hypnotics are available in primary care,^39^ many organisational and individual barriers were reported by both GPs and pharmacists. The main barriers were a lack of time and motivation due to few success stories and many other public healthcare problems to address. Also, knowledge and skills regarding deprescribing need improvement. This largely corresponds to previous research which found that a lack of knowledge about BZRAs by the prescriber was a barrier to discontinuation,^44^ as was the fear of withdrawal syndrome in patients.^47^ Interprofessional collaboration could play an important role in addressing these barriers, due to the complementarity in knowledge of GPs and pharmacists, and an increase in support for both the professionals and the patients when deprescribing.^48^ Previous research also showed that inappropriate BZRA use can decrease up to 50% in a collaborative primary care setting.^49^

Finally, although many theory‐based interventions for primary care could be developed based on this study, community actions are particularly interesting because they could help build a supportive context for reducing long‐term BZRA use. Altering public views on hypnotic use, on patients' capabilities to handle stress, and on healthcare as more than providing medication, seem promising because they could alter the basic premises that currently steer treatment decisions. Moreover, community interventions have been found to increase self‐care in patients with chronic health problems.^50^

### 4.1 Strengths and limitations

To the best of our knowledge, this is the first study to map the reflexivity between factors at different levels, including the impact of societal beliefs, that contribute to long‐term BZRA use for insomnia in primary care. The strengths of this study lie in the grounded theory approach, which enabled the integration of existing knowledge with the collected data. Furthermore, using in‐depth interviews to explore a phenomenon with three key stakeholders in primary care provided a rich data set for the development of themes. Another strength lay in the analytical approach and intermittent team discussions with a multidisciplinary team with different levels of research experience, which facilitated bracketing^51^ during analysis. Concurrently, data interpretation is a part of qualitative research, which may enable transfer to similar contexts, but not universal generalisability. Although rare, there are two advantages to having used the TDF solely for data interpretation. First, it allowed for a flexible data collection process, more specifically with a grounded theory approach. Second, the results better inform the development and implementation of new interventions than a general thematic analysis would have done. Finally, the study was limited because we interviewed a convenience sample, which may have produced biased data.

## 5 CONCLUSION

Long‐term BZRA use for insomnia is a complex and multifaceted public health problem that is not adequately addressed in primary care at this time. Although primary care professionals in this study found discontinuation of long‐term BZRA use relevant to the patient's health, many organisational and personal barriers were reported. The current social and healthcare context is not empowering for patients and professionals to reduce long‐term BZRA use for insomnia. Specifically, for primary care, all stakeholders reported the need for a nonmedicalised relationship between the patient and GP to lower prescribing rates. Thus, the implementation of new interventions or guidelines to reduce long‐term BZRA use for insomnia in primary care should be combined with other initiatives for the management of insomnia. After all, patient safety cannot come at the expense of proper treatment.

## AUTHOR CONTRIBUTIONS

**Kristien Coteur**: Conceptualisation (lead); investigation (lead); formal analysis (equal); methodology (supporting); writing—original draft preparation (lead); writing—review and editing (equal). **Sanne Peters**: Formal analysis (equal); methodology (lead); writing—original draft preparation (supporting); writing—review and editing (equal). **Pieter Jansen**: Formal analysis (equal); writing—original draft preparation (supporting); writing—review and editing (equal). **Birgitte Schoenmakers**: Formal analysis (supporting); methodology (supporting); supervision (supporting); writing—original draft preparation (supporting); writing—review and editing (equal). **Marc Van Nuland**: Conceptualisation (supporting); formal analysis (equal); methodology (supporting); supervision (lead); writing—original draft preparation (supporting); writing—review and editing (equal).

## CONFLICT OF INTEREST STATEMENT

The authors declare no conflict of interest.

## ETHICS STATEMENT

Ethics approval was obtained from the Social and Societal Ethics Committee of KU Leuven in August 2021 (G‐2021‐3713‐R2(MAR)). All participants provided written informed consent before the interview.

## Supporting information

Supporting information.

## Data Availability

The data that support the findings of this study are available from the corresponding author upon reasonable request.
