# Supplementary material for: Reducing long‐term use of benzodiazepine receptor agonists: In‐depth interview study with primary care stakeholders
Source: Health Expect. 2023 Oct 17;27(1):e13888. doi: 10.1111/hex.13888 (PMC10726152; doi:10.1111/hex.13888)
Supplement: Supplementary file 1 — Supporting information. [file HEX-27-e13888-s001.pdf]

APPENDIX 1. CODING TREE

Translated from Dutch to English using deepl.com;

For both confirmation and denial of a phenomenon the same code was used.

Name	Description	Files	Refs
Other factors	factors that contribute to why BZRA are used for insomnia, but do not immediately translate to a need, sometimes opposite of a need, e.g. need apo = good working relationship with physician; other factors that may trigger (or inhibit) change = poor working relationship with physician (from real-life situations)	0	0
meso~ GPs and pharmacists practice		1	1
context	contextual factors of the professional that influence prescribing behavior	11	28
Knowledge and skills		13	40
Learning from peer experience		2	2
Training regarding sleep, alternatives and sleep medication		11	16
Personal motivation		9	35
Low return on investment		3	3
Don’t want to keep repeating yourself	behind this is the need for a good relationship with the patient; see needs to discuss use/abandonment	5	5
Do not want to impose		3	4
Feel personally responsible		14	36
Professional network for referral	Need network/resources to refer when needed; refer to other professionals who can e.g. make more time	5	10
collaboration~prof. domain		11	19
pharm does not know indication for benzo or Z	excerpts indicating that it is sometimes difficult to speak with the patient because of not knowing underlying diagnosis	4	5
Referral to other professional	Who, for example, can make more time	1	1
Power physician	ties in closely with accountability, but here it is more about control: the physician decides on the patient's treatment	13	21

Name	Description	Files	Refs
mandatory delivery by pharmacist		11	23
poor working relationship with the physician		4	5
lack of communication and explanation		7	10
caregiver misunderstandings		1	2
responsibility physician	here is about how the physician is responsible for treating the patient correctly: tailored to what is needed; prescriptions are their responsibility	22	95
power, impact pharmacist	examples where it becomes clear that the pharmacist also has power/impact despite mandatory dispensing and despite the power and responsibility of the physician; e.g., apo addressed physician on prescriptions for a patient whose use was becoming irresponsible; physician did not change anything, then apo felt entitled to intervene and start counseling the patient in tapering off	14	28
give the same advice		9	22
micro~ patients		0	0
availability (sports and unsports) infrastructure		1	1
input decision process	this is about the magnitude of the patient's input: (examples of) resistance, etc.; when it is about a health care provider expressing how important it is for the patient to make the decision about his treatment, it rather fits with needs of health care providers: patient must make the choice	16	46
attitude toward psychologist		5	6
patient's choice		16	50
medication use		9	14
usage is running out of steam	at the discretion of the health care provider; these include brief examples of shoppers, as well as other inappropriate use	12	14
shopping behavior		6	8

Name	Description	Files	Refs
tacit approval by physician and pharm	it is not brought up so must be okay for physician and pharmacist; depends on patient need - being adequately informed, does occur frequently	5	6
access psychotherapy		3	5
financial~ price too high		2	3
fixed number of sessions while problem is very individual		1	1
waiting lists		1	1
addiction issues	when it is discussed that addiction plays an important role, or referred to other addictions requiring customized treatment, or influence of addiction susceptibility patients etc.	11	47
Ideas<br><br>*These codes will be discussed in a separate paper and are therefore not elaborated on in this coding tree.*	*concrete ideas that may influence motivation to use BZRA for insomnia*	1	2
needs		0	0
meso~ GPs and pharmacists practice		0	0
(effective) alternatives		12	50
why phytotherapy	any info on why or when phytotherapy is useful or not, anything that expresses needs about this	21	56
financial~livelihood		1	2
revenue pharm VS voluntary time commitment		7	11
revenue physician VS phasing out or not prescribing		4	6
patient health central		17	30
contraindications ~ interactions = review		6	8
help in acute situation		12	28
patient ~depanish~ without prescription	Examples demonstrating when/why a patient does or does not take medication without a prescription	3	4

Name	Description	Files	Refs
relationship with the patient	Statements substantiating that the pharm or physician knows their patient	9	11
always work on a tailored basis		11	38
stable (use) = continue		10	19
fall prevention = switching		7	9
improvements after winding down or stopping		1	5
good working relationship with the physician		10	16
Not wanting to give patient ~too much~ advice because may lead to not following doctor's appointments	Fear pharmacist	4	4
Do not want to undermine doctor-patient relationship	always feedback to the physician himself, not through the patient = do not question prescription in front of patient	2	2
Feedback to physician if (physician-planned) phaseout does not go as desired		7	8
prescribe smaller boxes	if it is expressed that smaller boxes may or may not be necessary/meaningful = here, even if it talks about prescribing tailored to the patient (e.g., 14 pills); if it is described as a possible change without a real need behind it, then at ideas for policy	10	20
patient motivation	In what way can/will the patient's motivation (be) influenced? = why would a patient consider changes	17	27
Empowering during wind-down with	you may also interpret this more broadly as education and discussion; materials/skills deemed necessary than	6	8
be available for questions		3	4
concern~involvement progress		7	10
repetition		7	14
Structural follow-up		12	44

Name	Description	Files	Refs
magistral preparation	If the tablets are difficult to break or for convenience of patient	9	16
medication schedule		13	32
visual materials		5	11
self-management		14	33
non-medicalized or prescription-based relationship between patient and physician	referrals to strong medicalization or contacts with family doctor because of need for prescription instead of need for care/listened ear/ etc.	10	18
Normalizing grief and other psychosocial processes		9	17
quick and inexpensive solutions		6	9
easiness prescription		10	19
taboo		2	2
To make use~decrease negotiable		9	19
attention drawn to it from outside~patient		16	24
Physician asks pharm to discuss tapering		3	4
campaign~ week of sleep = clue		3	3
dose increase		1	2
patient starts talking about it himself		19	39
patient falls or injury		12	22
expertise~knowledge insomnia		11	38
Shortage in pharm		3	5
good (long) relationship with the patient		16	37
Be able to express concern for patient health		7	15

Name	Description	Files	Refs
listening carefully to the patient		13	26
don't want to repeat yourself		10	20
no confrontation with patient		11	18
less stigma -~ open and honest communication possible		9	15
improved working conditions		2	4
time & space for medication reviews, psychosocial consultations		14	36
patient must make the (informed) choice about management of insomnia	even if it is about starting or discontinuing medication, it may be below, i.e., expressions that clarify that doctor and apo believe the choice is up to the patient	12	62
rehabilitation hospital, residential care center, etc.		8	15
micro~ patients		0	0
financially		9	13
cheapest solution		5	7
wins from antibiotics, priorities in conflict with health		1	1
daytime functioning		5	12
life adjustments		1	3
daytime functioning or comfort		17	56
life adjustments		8	21
Hold (on to/grip)		7	13
~this is with what I'm good, no adjusting~		11	16
have in house for ~emergencies~		6	8

Name	Description	Files	Refs
often deep sorrow or complex situation behind		9	16
need for rest	as a motive	16	40
To make use~decrease discussable		7	11
(no) taboo		20	53
expertise	maybe they would rather discuss it with the doctor than the pharmacist?	10	32
privacy and safe environment		8	12
respectful communication		12	35
recognition of complex problems	Need for recognition of problems leading to sleep drug use	8	20
problem recognition		1	3
peers		10	53
triggers	issues that drove people to wind down or discuss winding down; correlates with codes around input decision process and patient choice	6	19
follow-up	by physician and/or pharmacist	0	0
Being able to hear partner at night - in old age	need to be able to follow up with partner (whose health is worse), which would not go with taking sleep medication	1	2
Social support	Vb. Social network support provided by non-profit organization LUS	5	11
be sufficiently informed		14	66
alternatives		12	61
health effects	such as dementia, or other effects that make the patient think	10	22
information sources		12	56
group sessions		10	61
caregivers		2	3
online tools		12	32

Name	Description	Files	Refs
already used yourself		5	6
side effects		15	40
follow the schedule		13	34
adjustments possible, indicate own pace		11	30
addictive effect		14	56
effect of sleep and sleep medication		1	6
